# Superior Acetone Selectivity in Gas Mixtures by Catalyst‐Filtered Chemoresistive Sensors

**DOI:** 10.1002/advs.202001503

**Published:** 2020-08-16

**Authors:** Ines C. Weber, Hugo P. Braun, Frank Krumeich, Andreas T. Güntner, Sotiris E. Pratsinis

**Affiliations:** ^1^ Particle Technology Laboratory Department of Mechanical and Process Engineering ETH Zurich Sonneggstrasse 3 Zurich 8092 Switzerland

**Keywords:** breath analysis, environmental monitoring, semiconductors, solid‐state gas sensors, wearables

## Abstract

Acetone is a toxic air pollutant and a key breath marker for non‐invasively monitoring fat metabolism. Its routine detection in realistic gas mixtures (i.e., human breath and indoor air), however, is challenging, as low‐cost acetone sensors suffer from insufficient selectivity. Here, a compact detector for acetone sensing is introduced, having unprecedented selectivity (>250) over the most challenging interferants (e.g., alcohols, aldehydes, aromatics, isoprene, ammonia, H_2_, and CO). That way, acetone is quantified with fast response (<1 min) down to, at least, 50 parts per billion (ppb) in gas mixtures with such interferants having up to two orders of magnitude higher concentration than acetone at realistic relative humidities (RH = 30–90%). The detector consists of a catalytic packed bed (30 mg) of flame‐made Al_2_O_3_ nanoparticles (120 m^2^ g^−1^) decorated with Pt nanoclusters (average size 9 nm) and a highly sensitive chemo‐resistive sensor made by flame aerosol deposition and in situ annealing of nanostructured Si‐doped *ε*‐WO_3_ (Si/WO_3_). Most importantly, the catalytic packed bed converts interferants continuously enabling highly selective acetone sensing even in the exhaled breath of a volunteer. The detector exhibits stable performance over, at least, 145 days at 90% RH, as validated by mass spectrometry.

## Graphical abstract

A solid‐state detector is presented that quantifies acetone down to 50 parts per billion (ppb) with unmet selectivity in gas mixtures having two orders of magnitude higher interferant concentrations and in exhaled human breath. It is low‐cost, combining a nanostructured Pt/Al_2_O_3_ catalyst with a Si/WO_3_ chemoresistive sensor in a compact design, and is promising for hand‐held breath analyzers or wearable air quality monitors.

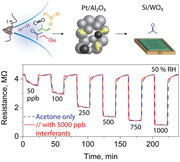

## 1 Introduction

Acetone is a major industrial chemical (6.2 Mt per year^[^ ^1^ ^]^) but also a toxic^[^ ^2^ ^]^ air pollutant. In addition, it is a proven breath marker for tracking non‐invasively fat metabolism,^[^ ^3^ ^]^ making its accurate detection necessary for breath analysis during exercise^[^ ^4^ ^]^ and dieting^[^ ^5^ ^]^ as well as search and rescue.^[^ ^6^ ^]^ So far, several techniques have been applied for online^[^ ^4^ ^]^ and offline (e.g., with Tedlar bags^[^ ^7^ ^]^) acetone detection, including gas chromatography,^[^ ^8^ ^]^ mass spectrometry,^[^ ^9^ ^]^ and chemical adsorption columns.^[^ ^10^ ^]^ However, these are usually expensive,^[^ ^11^ ^]^ non‐portable,^[^ ^9^ ^]^ require trained personnel, or are single‐use only.^[^ ^10^ ^]^ To date, this prevents easy, routine, and widespread acetone monitoring.

Modern solid‐state sensors based on chemoresistive nanostructured materials (e.g., hollow Pt/WO_3_ nanotubes,^[^ ^12^ ^]^ Au‐decorated vertical hematite nanotubes,^[^ ^13^ ^]^ or Ni/ZnO^[^ ^14^ ^]^) bear high potential for compact or even hand‐held acetone analyzers due to their high sensitivity,^[^ ^15^ ^]^ low power consumption,^[^ ^16^ ^]^ high miniaturization potential,^[^ ^17^ ^]^ and simplicity.^[^ ^18^ ^]^ Despite extensive research on such sensors, a bottleneck remains their insufficient selectivity (**Table** **1**) in real gas mixtures with hundreds of compounds (like breath^[^ ^19^ ^]^ or indoor air^[^ ^20^ ^]^) and high RH.^[^ ^21^ ^]^ More specifically, ethanol from cleaning agents and disinfectants can reach hundreds of parts per million (ppm)^[^ ^22^ ^]^ in clinical settings or gyms, exceeding average breath acetone concentrations (477 ppb mean^[^ ^9^ ^]^) by orders of magnitude. Another interferant is exhaled endogenous H_2_ from bacterial decomposition of carbohydrates occurring at up to 188 ppm in the breath of humans with lactose‐intolerance after food intake.^[^ ^23^ ^]^ Finally, other analytes like isoprene that can spike up to 400 ppb^[^ ^24^ ^]^ during physical activity have rarely been considered but can be problematic as well (e.g., for Si/WO_3_, Table 1).

**Table 1:** Selectivity comparison of solid‐state acetone sensors (*this work)

Material			RH [%]	Selectivity, *S*_acetone_/*S*_analyte_ [—]					Ref.
				Ethanol	Isoprene	H_2_	CO	Ammonia	
Optical		La_2_O_3_ cataluminescence	0	12.5	—	—	—	>1000	^[^ ^25^ ^]^
Chemo‐resistive	Composites	SnO_2_ / reduced graphene oxide	90	4.3	—	—	9.5	9.5	^[^ ^26^ ^]^
		SnO_2_ / multi‐walled carbon nanotubes	0	1.8	—	—	2.9	—	^[^ ^27^ ^]^
		Ni‐SnO_2_ / graphene	0	1.9	—	18	—	—	^[^ ^28^ ^]^
	Metal Oxides	ZnFe_2_O_4_ nanospheres	0	2.25	—	—	54	—	^[^ ^29^ ^]^
		PdO/Co_3_O_4_ nanocages	90	2.2	—	—	—	5.5	^[^ ^30^ ^]^
		Co/ZnO nanofibers	25	4.7	—	12	150	25	^[^ ^31^ ^]^
		Al/ZnO	0	—	3.6	—	7.4	6.9	^[^ ^32^ ^]^
		Ni/ZnO	40	3.6	—	49	970	—	^[^ ^14^ ^]^
		Au‐vertical hematite nanotubes	50	2.4	—	87	86	36	^[^ ^13^ ^]^
		*γ*‐WO_3_	65	6.8	—	88	—	29	^[^ ^33^ ^]^
		Pt/WO_3_ nanotubes	85	6.3	—	—	—	—	^[^ ^12^ ^]^
		Faceted h‐WO_3_	25	8.7	—	—	—	24.7	^[^ ^34^ ^]^
		TiO_2_/WO_3_ nanocrystals	40	20	—	14	—	—	^[^ ^35^ ^]^
		Cr/WO_3_	0	6.7	—	—	200	10	^[^ ^36^ ^]^
		Si/WO_3_	90	11.4	0.5	139	289	140	*
		ZnO + Si/WO_3_	90	81	—	—	—	—	^[^ ^37^ ^]^
		Pt/Al_2_O_3_ + Si/WO_3_	90	>500	>1000	>250	>1000	>1000	*

Catalytic filters upstream of the sensor can improve selectivity by continuously converting interferants to less reactive or inert species,^[^ ^38^ ^]^ as established for propane^[^ ^39^ ^]^ or methane^[^ ^38^ ^]^ sensing. Particularly compact are catalytic overlayers directly deposited onto the sensing films (e.g., Cr_2_O_3_ on SnO_2_ for ethylene in food monitoring^[^ ^40^ ^]^). This approach is advantageous over physical sorption filters (e.g., activated alumina^[^ ^41^ ^]^ or Tenax packed beds^[^ ^42^ ^]^) that saturate and require cleaning. Also molecular sieves (e.g., microporous zeolite membranes^[^ ^43^ ^]^ or graphene oxide layers^[^ ^44^ ^]^) are not suitable since acetone (4.7 Å^[^ ^45^ ^]^) features little size difference, for instance, to ethanol (4.3 Å^[^ ^45^ ^]^) or isoprene (5.5 Å^[^ ^46^ ^]^). More recently, a catalytic packed bed removed ethanol selectively over acetone by exploiting the surface basicity of flame‐made ZnO particles.^[^ ^37^ ^]^ However, ZnO did not remove H_2_, isoprene, benzene, methanol, or toluene.^[^ ^37^ ^]^ As a result, none of these filters provides a suitable solution to selectively remove all critical interferants over acetone.

Here, a low‐cost, highly sensitive, and selective acetone detector is introduced combining a catalytic packed bed of Pt‐decorated Al_2_O_3_ (Pt/Al_2_O_3_) nanoparticles with a chemoresistive Si/WO_3_ sensor. The Al_2_O_3_ was selected as it preferentially reacts with alcohols (e.g., 2‐propanol) and even aromatics (e.g., toluene) rather than acetone,^[^ ^37^ ^]^ while Pt was added since it oxidizes H_2_ already at room temperature.^[^ ^47^ ^]^ The Si/WO_3_ was chosen due to its distinct acetone sensitivity to detect even the lowest traces (e.g., 20 ppb), with fast response times (i.e., 1.3 min at 100 ppb) and remarkable selectivity at high RH,^[^ ^48^ ^]^ superior to most other acetone sensing materials (Table 1). Also, it has proven performance in breath analysis during exercising^[^ ^4^ ^]^ and dieting^[^ ^49^ ^]^ with several human subjects. First, the detector performance was explored with acetone (down to 50 ppb), in the presence of ethanol, H_2_, isoprene, ammonia, CO, methanol, formaldehyde, acetaldehyde, toluene, and m‐xylene at 0–90% RH and their mixtures followed by benchmarking to state‐of‐the‐art acetone sensors. Second, the Pt/Al_2_O_3_ packed bed catalyst was characterized by X‐ray diffraction (XRD), energy dispersive X‐ray spectrometry (EDXS), N_2_ adsorption, transmission electron microscopy (TEM), and proton transfer reaction time‐of‐flight mass spectrometer (PTR‐ToF‐MS) to validate the sensor measurements. In addition, the detector was tested on the exhaled breath of one volunteer and its stability assessed over 145 days.

## 2 Results and Discussion

### 2.1 Acetone Selectivity and Benchmarking to State‐of‐the‐Art Sensors

**Figure 1:**
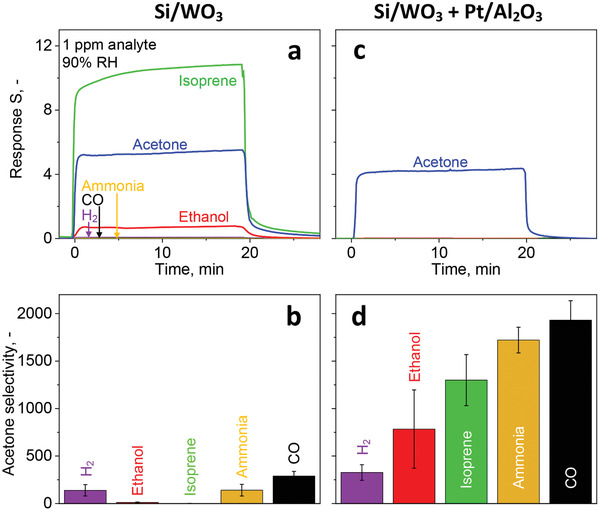
Si/WO_3_ sensor response to 1 ppm isoprene (green), acetone (blue), ethanol (red), H_2_ (purple), ammonia (yellow), or CO (black) in air at 400 °C and 90% RH after passing through the catalytic Pt/Al_2_O_3_ packed bed at a) room temperature (i.e., inactive) and c) at 135 °C. b,d) Corresponding acetone selectivities, as measured with the sensor. Error bars indicate the standard deviations for three identically prepared packed beds and sensors.

**Figure** **1**a shows the responses (i.e., ratio of film resistance in air [*R*_air_] over that with analyte [*R*_analyte_]: *S* = *R*_air_ / *R*_analyte_ − 1) of the Si/WO_3_ sensor at 400 °C upon exposure to 1 ppm of isoprene, acetone, H_2_, ammonia, CO, or ethanol at 90% RH after passing through the Pt/Al_2_O_3_ bed at room temperature (i.e., inactive). The sensor responds rapidly to each analyte (e.g., 20 s for acetone). However, it cannot distinguish acetone (*S* = 5.4) from isoprene (*S* = 10.4) and is also interfered by ethanol (*S* = 0.7). The corresponding acetone selectivities for three identically prepared sensors (*S*_acetone_/*S*_analyte_, Figure 1b) range from 0.5 ± 0.07 (isoprene) to 290 ± 50 (CO) and are in agreement with literature^[^ ^48^ ^]^ for ethanol (12.7 ± 4.0).

**Figure 2:**
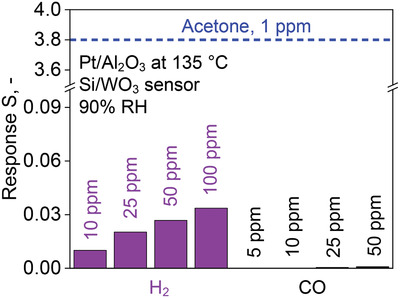
Responses of the Si/WO_3_ sensor with catalytic Pt/Al_2_O_3_ packed bed at 135 °C upon exposure to 5, 10, 25, 50, and 100 ppm of H_2_ (purple) and CO (black) at 90% RH. The response to 1 ppm acetone is indicated (blue dashed line) for reference. Note the scale break of the ordinate.

When, however, the Pt/Al_2_O_3_ packed bed is kept at 135 °C upstream of the sensor, only acetone is detected (Figure 1c). In fact, the acetone response is reduced only by about 20% to 4.3 while the response to all other analytes is below 0.0015 indicating their practically full conversion by Pt/Al_2_O_3_. This results in unprecedented high acetone selectivities: over 250 for H_2,_ above 1000 for isoprene and ammonia, and nearly 2000 for CO (Figure 1d). This excellent acetone selectivity is maintained even at orders of magnitude higher and realistic interferant concentrations (e.g., up to 100 ppm H_2_^[^ ^23^ ^]^ and 50 ppm CO^[^ ^50^ ^]^ in **Figure** **2**) that can be present in exhaled breath. Most importantly, the packed bed hardly affects the fast response time of the sensor (please compare Figure 1a vs 1c). Such high selectivities outperform state‐of‐the‐art acetone sensors (Table 1). It is also superior to packed beds of ZnO nanoparticles^[^ ^37^ ^]^ (at 260 °C), that left H_2_ and roughly half (45%) of isoprene intact. In fact, the best acetone selectivities of such sensors to ethanol ≤81^[^ ^35^ ^]^ and isoprene ≤3.6^[^ ^32^ ^]^ are overcome by the present detector by 10 and 350 times, respectively. Remarkable selectivities had been achieved already for H_2_,^[^ ^33^ ^]^ CO,^[^ ^14^ ^]^ and/or ammonia,^[^ ^14^ ^]^ but even these are improved consistently by the present detector.

### 2.2 Selective Acetone Detection at ppb in Gas Mixtures

**Figure 3:**
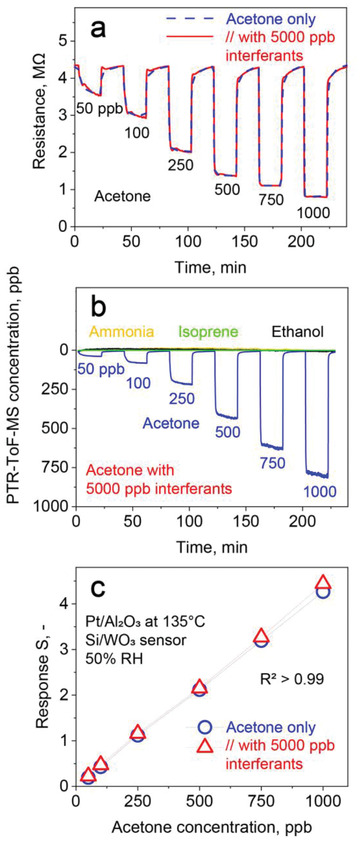
a) Si/WO_3_ film resistance upon exposure to 50–1000 ppb acetone as single analyte (blue dashed line) and with a mixture containing simultaneously 5000 ppb ammonia, isoprene, ethanol, CO, and H_2_ (each 1000 ppb, red solid line) at 50% RH after passing through the Pt/Al_2_O_3_ packed bed at 135 °C. b) Analyte concentration profiles of the same gas mixtures after the Pt/Al_2_O_3_ packed bed, as measured by PTR‐ToF‐MS that, however, cannot detect CO and H_2_. c) Acetone sensor responses without (circles) and with the above mixture of interferants (triangles). The overlapping symbols and high coefficient of determination (*R* ^2^ > 0.99, *n* = 6) highlight the excellent acetone selectivity of the present detector.

The performance of the detector is shown best with gas mixtures of relevant acetone concentrations with interferants at high concentrations. **Figure** **3**a depicts the Si/WO_3_ sensor resistance upon exposure to 50–1000 ppb acetone at 50% RH (dashed blue line). The sensor resistance drops immediately when exposed to acetone, for instance, from 4.34 to 3.66 MΩ for 50 ppb with high signal to noise ratio (SNR, >100). The extrapolated lower limit of detection (LOD, at SNR = 3) is 5.5 ppb, competitive to more complex gas chromatography‐ion mobility spectrometry (GC‐IMS, 30 ppb for acetone^[^ ^51^ ^]^). After each exposure, the baseline is recovered, making the detector suitable for frequent measurements.

Most importantly, when testing the same acetone concentrations in the presence of 1000 ppb of ethanol, isoprene, ammonia, H_2_, and CO (a total of 5000 ppb of all these interferants), the sensor response does not change (solid red line). A magnification exemplarily for 250 ppb acetone is shown in Figure S1, Supporting Information. This indicates that all interferants are converted in the Pt/Al_2_O_3_ packed bed, as confirmed for ammonia, isoprene and ethanol by PTR‐ToF‐MS (Figure 3b), highlighting the excellent selectivity of the detector. Also, fast response and recovery times of 55 and 100 s (at 500 ppb acetone) of the detector for such low acetone concentrations are preserved when sampling gas mixtures, as required for air quality monitoring and online breath analysis (e.g., with buffered^[^ ^52^ ^]^ end‐tidal breath sampling^[^ ^53^ ^]^).

The corresponding responses to 50–1000 ppb acetone without (circles) and with (triangles) 5000 ppb interferants are shown in Figure 3c. The sensor response increases linearly with concentration, in line with linear diffusion‐reaction theory^[^ ^48^ ^]^ at such low concentrations. The average deviation between single acetone and its mixtures with interferants in the breath relevant range (150–1000 ppb) is only 3%.

### 2.3 Pt/Al_2_O_3_ Material Characterization

**Figure 4:**
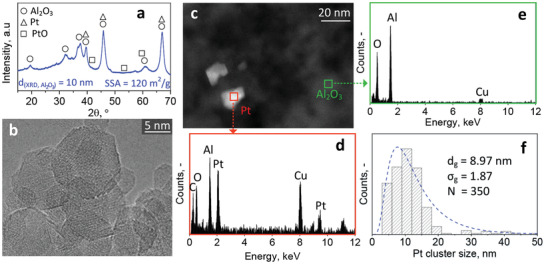
a) XRD pattern of 0.2 mol% Pt/Al_2_O_3_ powder with reference peaks for cubic Al_2_O_3_ (circles), Pt (triangles), and tetragonal PtO (squares). The Al_2_O_3_ crystal size (*d*_XRD_) and SSA are indicated. b) HRTEM image of such crystalline Pt/Al_2_O_3_ particles. c) HAADF‐STEM of Al_2_O_3_ particles (dark grey) and Pt clusters (white). EDXS analysis of d) a Pt cluster and e) Al_2_O_3_ support, as indicated in (c). f) Counted Pt cluster size distribution, as determined from HAADF‐STEM images. Equivalent lognormal fit from the counted distribution (dashed line) together with its geometric diameter (*d*_g_), standard deviation (*σ*_g_), and number count of clusters (*N*).

Next, the characteristics of Pt/Al_2_O_3_ were investigated to better understand its acetone selectivity, while this had been done for the Si/WO_3_ sensor elsewhere.^[^ ^48^, ^54^ ^]^ The XRD pattern of annealed 0.2 mol% Pt/Al_2_O_3_ is shown in **Figure** **4**a with reference peak positions indicated by symbols. Distinct peaks indicate cubic *γ*‐Al_2_O_3_ (circles) with crystal size of 10 nm. Since the average particle size is 12.6 nm (as determined by N_2_ adsorption), monocrystallinity appears dominant, in agreement with literature.^[^ ^55^ ^]^ Neither cubic Pt (triangles) nor tetragonal PtO (squares) crystal reflections are observed that become visible only for Pt loadings at and above 1 mol% (Figure S2, blue and green patterns, Supporting Information).

Figure 4b shows a high resolution transmission electron microscopy (HRTEM) image of such Al_2_O_3_ nanoparticles having rather spherical shape with sizes ranging from 5 to 20 nm, in line with XRD and N_2_ adsorption measurements (Figure 4a). Visible lattice fringes confirm crystallinity. Clear distinction of the metallic Pt particles/clusters from the Al_2_O_3_ support is obtained with high angle annular dark field scanning transmission electron microscopy (HAADF‐STEM, Figure 4c). The Pt particles appear brighter due to their higher scattering potential (Z contrast). They form faceted and non‐spherical crystalline clusters, in line with HRTEM (Figure S3, Supporting Information). In fact, EDXS (Figure 4d) of a Pt cluster (Figure 4c, red square) indicates the presence of Pt as well as Al and O. In contrast, Al_2_O_3_ appears darker, as confirmed by EDXS analysis where no Pt is detected (Figure 4e and green square in Figure 4c). The peaks of Cu and C originate from the perforated TEM grid.

Furthermore, STEM reveals Pt particle aggregation (Figure S4a–d, Supporting Information), that likely stems from sintering^[^ ^56^ ^]^ during the heat treatment for 1 h at 500 °C. The size distribution for 350 Pt particles or clusters from counting HAADF‐STEM images is shown in Figure 4f. A log‐normal distribution (dashed line) is obtained, with a geometric average diameter (*d*_g_) and standard deviation (*σ*_g_) of 8.97 nm and 1.87, respectively.

### 2.4 Catalytic Characterization and Conversion Mechanism

**Figure 5:**
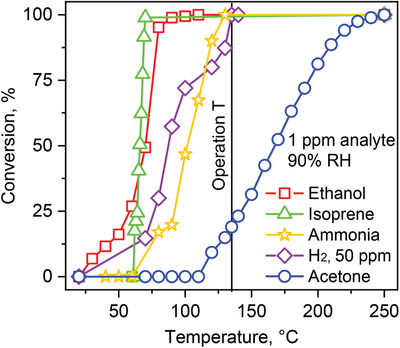
Catalytic conversion of 1 ppm ethanol (squares), isoprene (triangles), ammonia (stars), H_2_ (diamonds, 50 ppm), and acetone (circles) over 0.2 mol% Pt/Al_2_O_3_ at 90% RH as a function of catalytic packed bed temperature. All analyte concentrations were measured at the catalyst outlet by PTR‐ToF‐MS, except for H_2_ where a QuinTron Breath Tracker was used.

The catalytic performance of the packed bed of 0.2 mol% Pt/Al_2_O_3_ nanoparticles was investigated on the conversion of 1 ppm ethanol (squares), isoprene (triangles), ammonia (stars), acetone (circles), as well as 50 ppm H_2_ (diamonds) at 90% RH between 25 and 250 °C (**Figure** **5**) with a high resolution PTR‐ToF‐MS and a QuinTron Breath Tracker (for H_2_). Note that higher H_2_ concentrations were needed due to the insufficient detection limit and accuracy of the H_2_ detector (i.e., 1 ppm).^[^ ^57^ ^]^

All interferants are fully converted at 135 °C (i.e., packed bed operation temperature, vertical black line), validating the sensor results (Figure 1c). More specifically, isoprene, ethanol, ammonia, and H_2_ reach 100% conversion at 70, 90, 130, and 135 °C, respectively. Most remarkably, at 135 °C, the catalytic packed bed is capable to convert also various other molecules (**Table** **2**), including short‐chained alcohols (e.g., methanol), aldehydes (e.g., formaldehyde and acetaldehyde), and even aromatics (e.g., toluene and m‐xylene), representing other potential interferants in breath and indoor air. In contrast, acetone conversion starts only at 110 °C and reaches 100% conversion at 250 °C. At 135 °C, only little acetone is lost (i.e., 19%), in agreement with the sensor measurements (Figure 1c).

**Table 2:** Conversion of 1 ppm of various volatiles (or 50 ppm H_2_) flowing through a packed bed of 0.2 mol% Pt/Al_2_O_3_ at 135 °C and 90% RH, as measured with PTR‐ToF‐MS and QuinTron Breath Tracker (for H_2_)

	Molecule	Molecular formula	Conversion at 135 °C [%]
Alcohols	Methanol	CH_3_OH	100
	Ethanol	C_2_H_5_OH	100
Aldehydes	Formaldehyde	CH_2_O	100
	Acetaldehyde	C_2_H_4_O	100
Inorganics	H_2_	H_2_	100
	Ammonia	NH_3_	100
Aromatics	Toluene	C_7_H_8_	100
	m‐Xylene	C_8_H_10_	100
Ketones	Acetone	C_3_H_6_O	23.2

Pristine Al_2_O_3_ converts alcohols and even aromatics (e.g., toluene) preferentially over acetone.^[^ ^58^ ^]^ In fact, ethanol and H_2_ are converted before acetone at 90% RH (Figure S5a, Supporting Information). Apparently, Pt on the Al_2_O_3_ support (Figure 4) promotes analyte conversion, as confirmed at higher Pt contents (Figure S5c,d, Supporting Information) that led to even lower conversion temperatures than 135 °C. However, with increasing Pt content, the acetone selectivity over H_2_ deteriorates. So 0.2 mol% Pt was a compromise between selectivity and conversion temperature at minimal Pt content and was selected for the detector.

Alcohol oxidation on Pt/Al_2_O_3_ is promoted by surface‐adsorbed oxygen‐ or hydroxyl‐containing species, forming the intermediate acetaldehyde,^[^ ^59^ ^]^ as observed here as well on 0.2 mol% Pt/Al_2_O_3_ between 30 and 110 °C (Figure S6, Supporting Information). Also CO,^[^ ^60^ ^]^ formaldehyde,^[^ ^61^ ^]^ isoprene,^[^ ^61^ ^]^ and toluene^[^ ^61^ ^]^ interact with surface‐adsorbed hydroxyl groups on metal‐oxides. In contrast, acetone oxidation on metal‐oxide surfaces is typically promoted by coordinative binding^[^ ^62^ ^]^ to Lewis acid sites.^[^ ^63^ ^]^ Water molecules dissociate and chemisorb on such sites,^[^ ^64^ ^]^ reducing the surface reactivity for acetone at elevated RH. Most importantly, Al_2_O_3_ does not exhibit Brønsted sites that can oxidize acetone in humid environments,^[^ ^63^ ^]^ as observed for Nb_2_O_5_^[^ ^63^ ^]^ and WO_3_.^[^ ^65^ ^]^ In fact, WO_3_ oxidized acetone at almost identical temperatures as ethanol at 90% RH.^[^ ^37^ ^]^

### 2.5 Reality Check: Humidity Robustness, Breath Analysis, and Stability

**Figure 6:**
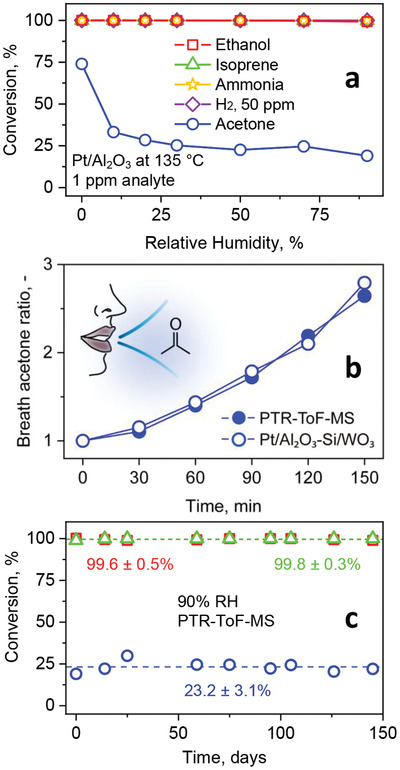
a) Conversion of 1 ppm ethanol (squares), isoprene (triangles), ammonia (stars), H_2_ (diamonds, 50 ppm), or acetone (circles) over 0.2 mol% Pt/Al_2_O_3_ at optimal 135 °C as a function of RH, as measured by PTR‐ToF‐MS and the QuinTron Breath Tracker. b) Breath acetone ratio (normalized to initial acetone concentration) after 40 min intense exercise^[^ ^4^ ^]^ by a single volunteer measured with the PTR‐ToF‐MS (filled symbols) and the Pt/Al_2_O_3_‐Si/WO_3_ detector (empty symbols) for *n* = 6 breath samples proving its superior selectivity. c) Packed bed stability for a mixture of 1 ppm ethanol, isoprene, and acetone over 145 days at 90% RH, as measured at the catalyst outlet by PTR‐ToF‐MS and the QuinTron Breath Tracker. Continuous variables are expressed as mean ± standard deviation (*n* = 9).

To investigate the detector performance in realistic conditions, its responses to varying humidity, breath conditions, and its long term stability were explored. First, the packed bed reactivity was examined at 135 °C by flowing through 1 ppm ethanol (squares), isoprene (triangles), ammonia (stars), H_2_ (diamonds, 50 ppm), or acetone (circles) at RH between 0% and 90% (**Figure** **6**a). All interferants are fully converted independent of RH. Most importantly, acetone conversion is rather stable (i.e., 23.1 ± 2.6%) between 30% and 90% RH, and in line with measurements in gas mixtures with 5000 ppb interferants (i.e., 19% acetone loss at 1000 ppb and 50% RH, Figure 3b), but drastically increases (i.e., 73.9%) in dry air, similar to ZnO.^[^ ^37^ ^]^ Therefore, the reactivity of Pt/Al_2_O_3_ is indeed reduced by RH, enabling the high acetone selectivity (Figure 5) and supporting further the above discussion on the deactivation of surface Lewis sites. Furthermore, it is important to highlight the robust packed bed performance between 30% and 90% RH. This is particularly useful as RH may vary in indoor air and exhaled human breath has up to 97% RH.^[^ ^66^ ^]^

Next, the detector was tested on exhaled human breath to demonstrate acetone selectivity in a gas mixture with hundreds of compounds.^[^ ^19^ ^]^ Figure 6b shows the breath acetone ratio of one volunteer for 150 min as measured with the present detector (Pt/Al_2_O_3_ packed bed followed by the Si‐doped WO_3_ sensor, open symbols) and the bench‐top PTR‐ToF‐MS (filled symbols). The volunteer had fasted overnight and performed 40 min exercise prior to the test to stimulate the fat metabolism.^[^ ^19^ ^]^ The steep acetone increase indicates enhanced body fat metabolism,^[^ ^67^ ^]^ as had been demonstrated before in exercise^[^ ^4^ ^]^ and dieting^[^ ^49^ ^]^ studies by both breath and blood analyses. Most remarkably, the detector quantifies correctly the acetone dynamics, in excellent agreement to PTR‐ToF‐MS. As a result, it is promising for personal breath analysis at home or in clinics though this needs to be confirmed with more volunteers.

Furthermore, the stability of the 0.2 mol% Pt/Al_2_O_3_ catalytic packed bed is investigated exemplarily for a mixture of 1 ppm acetone, isoprene, and ethanol at 135 °C and 90% RH over 145 days by PTR‐ToF‐MS (Figure 6c). It should be noted that the long term stability of flame‐made sensors has been established since 2006. Isoprene and ethanol are fully converted (>99.6%) over the entire time while only part of acetone is lost (23.2 ± 3.1%), in good agreement with the sensor measurement (Figure 1c). The stability of the catalytic performance indicates that the surface interaction of these analytes on Pt/Al_2_O_3_ is fully reversible maintaining its high reactivity. The XRD patterns of the fresh and spent Pt/Al_2_O_3_ (Figure S7, Supporting Information) indicate unaltered cubic Al_2_O_3_ crystal size (i.e., 10 nm), but some orthorhombic Al_2_O_3_ is formed. However, this does not compromise the catalytic performance in Figure 6c. This is a clear advantage over other nanoscale catalysts prone to growth even at room temperature (e.g., pure ZnO below 20 nm^[^ ^68^ ^]^) that are only applicable at larger particle sizes (e.g., 40 nm^[^ ^37^ ^]^), compromising the catalyst specific surface area available for conversion. Such stable performance is required for real‐life applications, where these catalytic packed beds will be applied on‐demand or even continuously over months.

Such detectors comprising catalytic packed beds and sensors are attractive for commercial devices. In fact, the sensor fabrication by flame aerosol technology is suitable for industrial‐scale production due to its compatibility with complementary metal‐oxide‐semiconductor (CMOS) processing.^[^ ^69^ ^]^ Also, the sensor and packed bed can be incorporated readily into compact and inexpensive devices with wireless smartphone communication, despite distinctly different operation temperatures, as shown with a pocket‐sized detector for sensing adulteration of alcoholic beverages.^[^ ^70^ ^]^

## 3 Conclusions

A highly sensitive and selective acetone detector was presented, combining a catalytic packed bed of Pt/Al_2_O_3_ nanoparticles with a nanostructured Si/WO_3_ chemo‐resistive sensor. This detector quantified acetone concentrations at high RH in gas mixtures with up to two orders of magnitude higher interferant concentrations, superior to previous sensors. The high selectivity (>250 to all tested analytes) was attributed mostly to the Pt/Al_2_O_3_. When heated to 135 °C, this catalytic bed continuously converted interfering molecules (including alcohols, aldehydes, aromatics, isoprene, ammonia, H_2_, and CO) over 145 days and with high robustness to 30–90% RH, as revealed by PTR‐ToF‐MS. The inherent selectivity of Pt/Al_2_O_3_ was associated to the Al_2_O_3_ support while the Pt clusters/particles shifted the analyte oxidation to lower (i.e., 135 °C) temperature.

Such assemblies of catalytic packed beds and sensors in series can be incorporated readily into compact and inexpensive devices. As such they are promising for low‐ppb acetone detection in life‐style applications like non‐invasive exercise^[^ ^4^ ^]^ and diet^[^ ^49^ ^]^ monitoring through breath analysis, as demonstrated even with a volunteer here. In a broad perspective, the combination of selective catalytic packed beds with sensitive sensors opens up exciting new opportunities to systematically design highly sensitive and selective but low‐cost sensors that are needed in medical diagnostics,^[^ ^21^ ^]^ air^[^ ^71^ ^]^ or food quality monitoring.^[^ ^72^ ^]^

## 4 Experimental Section

##### Pt/Al_2_O_3_ Packed Bed

The Pt/Al_2_O_3_ nanoparticles were produced by flame spray pyrolysis (FSP).^[^ ^73^ ^]^ The FSP precursor solution consisted of platinum(II) 2,4‐pentanedionate (Alfa Aesar, Germany, Pt ≥ 48 wt%) and aluminium(III) sec‐butoxide (Sigma‐Aldrich, Switzerland, 97%) in a mixture (7:3 by volume) of xylene (Sigma‐Aldrich, ≥ 98.5%) and acetonitrile (Alfa Aesar, 99%) to achieve a total metal concentration of 0.5 m and a product Pt content of 0.2, 1, and 3 mol%.^[^ ^56^ ^]^ Precursor solutions were injected through the FSP nozzle at 5 mL min^−1^ and dispersed with 5 L min^−1^ oxygen into a fine spray (pressure drop 1.6 bar). A ring‐shaped flamelet (1.2 L min^−1^ methane and 3.2 L min^−1^ oxygen) was used to ignite and sustain spray combustion. Particles were collected on a glass‐fiber filter (GF‐6 Albet‐Hahnemuehle, 257 mm diameter) at 50 cm above the burner by a vacuum pump (Seco SV 1025 C, Busch, Switzerland). The particles were removed from the filter with a spatula, sieved (250 *μ*m stainless steel sieve) to get rid of glass‐fiber residues, and annealed at 500 °C for 1 h in an oven (Carbolite Gero). The catalytic packed bed consisted of 30 mg of such nanoparticles in a quartz glass tube with 4 mm inner diameter, resulting in a packed bed length of 1.5 cm. Prior to application, the packed bed was checked visually for voids and placed inside a tube furnace (Nabertherm, P320).

##### Material Characterization

Particles were characterized by XRD with a Bruker AXS D8 Advance diffractometer, operated at 40 kV and 30 mA. The diffraction patterns were recorded at 2*θ* (Cu K_*α*_) between 15° and 70° with a step size and speed of 0.011° and 0.0057° s^−1^, respectively. The software Bruker Diffrac.eva V3.1 was used to identify crystal phases with reference parameters of cubic Al_2_O_3_ (PDF 10–0425), orthorhombic Al_2_O_3_ (PDF 88–0107), cubic Pt (PDF 01–1311), and tetragonal PtO (PDF 85–0714). Crystal sizes were calculated with the Rietveld refinement method, using the software TOPAS 4.2. The specific surface area (SSA) was measured by N_2_ adsorption (Micromeritics Tristar II Plus) using the Brunauer–Emmett–Teller (BET) method. The particle size for 0.2 mol% Pt/Al_2_O_3_ was calculated using the density of Al_2_O_3_ (3.95 g cm^−3^), while Pt was neglected due to its low content (i.e., 0.2 mol%). Prior to N_2_ adsorption, powder samples were degassed at 150 °C for 1 h under N_2_. For TEM, the nanoparticles were dispersed in ethanol. A few drops of their suspension were deposited onto a perforated carbon foil supported on a copper grid. HAADF‐STEM and EDXS were performed on an aberration‐corrected HD‐2700CS (Hitachi) instrument, operated at 200 kV. The size of non‐spherical Pt particles was obtained from HAADF‐STEM images by taking an average of the longest diameter and the diameter perpendicular to it (Figure S4c–f, Supporting Information). High resolution images were obtained with a double‐corrected microscope JEM‐ARM300F (GrandArm, JEOL), operated at 300 kV.

##### Sensing Tests

The sensor consisted of 10 mol% Si‐doped WO_3_ nanoparticles made by FSP^[^ ^48^ ^]^ and directly deposited onto interdigitated electrodes on Al_2_O_3_ substrates (15 mm × 13 mm × 0.8 mm, Electronic Design Center, Case Western Reserve University). This sensor was mounted on a Macor holder installed inside a Teflon chamber^[^ ^74^ ^]^ and heated to 400 °C^[^ ^48^ ^]^ by applying a constant voltage to a Pt heater (R&S HMC803, Germany) on the substrate's back side. The temperature was monitored with a resistance temperature detector on the front of the substrate. The Si/WO_3_ sensor was connected downstream of the Pt/Al_2_O_3_ packed bed and gas mixing setup with heated (55 °C) inert Teflon tubing to avoid analyte adsorption and water condensation.

The gas mixing setup (Figure 1 in ref. [37]) with high‐precision mass flow controllers (MFCs, Bronkhorst) dosed acetone (17.9 ppm), ethanol (9.8 ppm), isoprene (17.5 ppm), ammonia (10 ppm), H_2_ (1000 or 50 ppm), or CO (501.8 ppm) all in synthetic air (Pan Gas) into synthetic air (Pan Gas, Switzerland, C*_n_*H*_m_* and NO*_x_* ≤ 100 ppb) to achieve an overall flow rate of 150 mL min^−1^. Note that detector evaluation was carried out with the packed bed at room temperature (i.e., inactive) and at 135 °C (i.e., active). To have the desired RH, synthetic air was bubbled through a 125 mL glass vessel (Drechsel bottle, sintered glass frit, Sigma‐Aldrich) filled with ultrapure water (Milli‐Q A10, Merck, Switzerland) and monitored with a humidity sensor (SHT2x, Sensirion AG). Note that 50% instead of 90% RH was chosen for measurements in the gas mixtures of Figure 3 due to a limitation of the gas mixing setup at such high analyte concentrations. The Si/WO_3_ sensor resistance was determined using a multimeter (Keithley 2700). The sensor response (*S*) was calculated according to Equation (1)

(1) $$\begin{array}{c} S=\frac{R_{\mathrm{air}}}{R_{\mathrm{analyte}}}-1 \end{array}$$

where *R*_air_ and *R*_analyte_ are the sensing film resistances in air and during analyte exposure, respectively. The sensor response and recovery times were the times necessary to reach and recover 90% of the response resistance change, respectively.

##### Catalytic Characterization

Catalytic measurements were done with a setup described elsewhere.^[^ ^37^ ^]^ The Pt/Al_2_O_3_ packed bed connected to the gas mixing setup was flushed with synthetic air (Pan Gas) for, at least, one hour prior to the measurements to condition the catalyst surface with humid air and remove any contaminants. Additionally, also methanol (19.7 ppm), acetaldehyde (17.4 ppm), formaldehyde (7.2 ppm in N_2_), toluene (10.0 ppm), and m‐xylene (10.0 ppm) were tested (all in synthetic air, Pan Gas). The exhaust gas from the packed bed was analyzed with a PTR‐ToF‐MS 1000 (Ionicon, Austria). The PTR‐ToF‐MS drift voltage, temperature, and pressure at measurement were 600 V, 60 °C, and 2.3 mbar, respectively. The H_3_O^+^ ions served as primary ions and analyte concentrations were determined at mass‐to‐charge (*m/z*) ratios of 18.03 (ammonia^[^ ^75^ ^]^), 31.02 (formaldehyde^[^ ^76^ ^]^), 33.03 (methanol^[^ ^75^ ^]^), 45.03 (acetaldehyde^[^ ^75^ ^]^), 47.05 (ethanol^[^ ^76^ ^]^), 59.05 (acetone^[^ ^76^ ^]^), 69.07 (isoprene^[^ ^75^ ^]^), 93.06 (toluene^[^ ^75^ ^]^), and 107.16 (m‐xylene^[^ ^75^ ^]^). Prior to each measurement, the PTR‐ToF‐MS was three‐point‐calibrated with the above gas standards over the relevant range. For H_2_ measurements, gas samples were taken at the catalyst outlet with a 30 mL syringe (AlveoSampler Breath Test Syringe, QuinTron Inc., USA) and analyzed immediately with a QuinTron Breath Tracker. Calibration was performed with a standard (QuinGas‐3). Analyte conversion was calculated according to Equation (2)

(2) $$\begin{array}{c} C=100\%\times \left(1-\;\frac{\mathrm{Concentratio}n_{\mathrm{catalyst}\;\mathrm{outlet}}}{\mathrm{Concentratio}n_{\mathrm{catalyst}\;\mathrm{inlet}}}\right) \end{array}$$

For stability tests, the catalytic powder was stored in a glass vial in room air and installed as packed bed prior to each experiment.

##### Human Breath

One healthy and non‐smoking volunteer without metabolic and cardiovascular disease history participated. He was asked to consume a low‐carb meal for dinner and abstain from alcohol and exercise, at least, 24 h prior to the study. The study protocol consisted of an overnight fast (>8 h), a submaximal aerobic exercise intervention (40 min) starting at 8 a.m., and a post‐exercise rest (150 min). During exercise, the volunteer started cycling at 20% of the second ventilatory threshold (VT_2_) for 5 min. Thereafter, the cycling load was ramped with an increase of 10% VT_2_ every 5 min up 100% VT_2_.

Breath samples were extracted only during the post‐exercise rest every 30 min with a tailor‐made buffered end‐tidal breath sampler with a tube length of 375 mm and no flow restrictor, as described in detail elsewhere.^[^ ^53^ ^]^ The breath sampler was connected to the PTR‐ToF‐MS and the detector simultaneously through inert and heated (55 °C) Teflon tubing to avoid water condensation. To maintain a constant flow, a pump (150 mL min^−1^; Schwarzer Precision, Essen, Germany) was connected at the detector outlet. Prior to the study, the volunteer gave informed written consent. This study was approved by the Ethikkommission Nordwest‐ und Zentralschweiz (EKNZ 2019–02362).

##### Statistical Analysis

No pre‐processing of the data was done. Data of repeated measurements under same conditions were presented as mean ± standard deviation. The sample sizes (*n*) for each statistical analysis were indicated. Comparative datasets were assessed by calculating the coefficient of determination (*R* ^2^). Statistical analyses were performed using the software OriginPro 2018G (OriginLab Corporation, Massachusetts, USA).

## Conflict of Interest

The authors declare no conflict of interest.

## Supporting information

Supporting Information
