# Supplementary material for: Superior Acetone Selectivity in Gas Mixtures by Catalyst‐Filtered Chemoresistive Sensors
Source: Adv Sci (Weinh). 2020 Aug 16;7(19):2001503. doi: 10.1002/advs.202001503 (PMC7539217; doi:10.1002/advs.202001503)
Supplement: Supplementary file 1 — Supporting Information [file ADVS-7-2001503-s001.pdf]

© 2020 Wiley-VCH GmbH

ADVANCED SCIENCE Open Access

Supporting Information

for *Adv. Sci.*, DOI: 10.1002/advs.202001503

Superior Acetone Selectivity in Gas Mixtures by Catalyst-filtered Chemoresistive Sensors

Ines C. Weber, Hugo P. Braun, Frank Krumeich, Andreas T. Güntner, Sotiris E. Pratsinis*

Supporting Information

Superior Acetone Selectivity in Gas Mixtures by Catalyst-filtered Chemoresistive Sensors

Ines C. Weber, Hugo P. Braun, Frank Krumeich, Andreas T. Güntner, Sotiris E. Pratsinis*

Figures & Captions

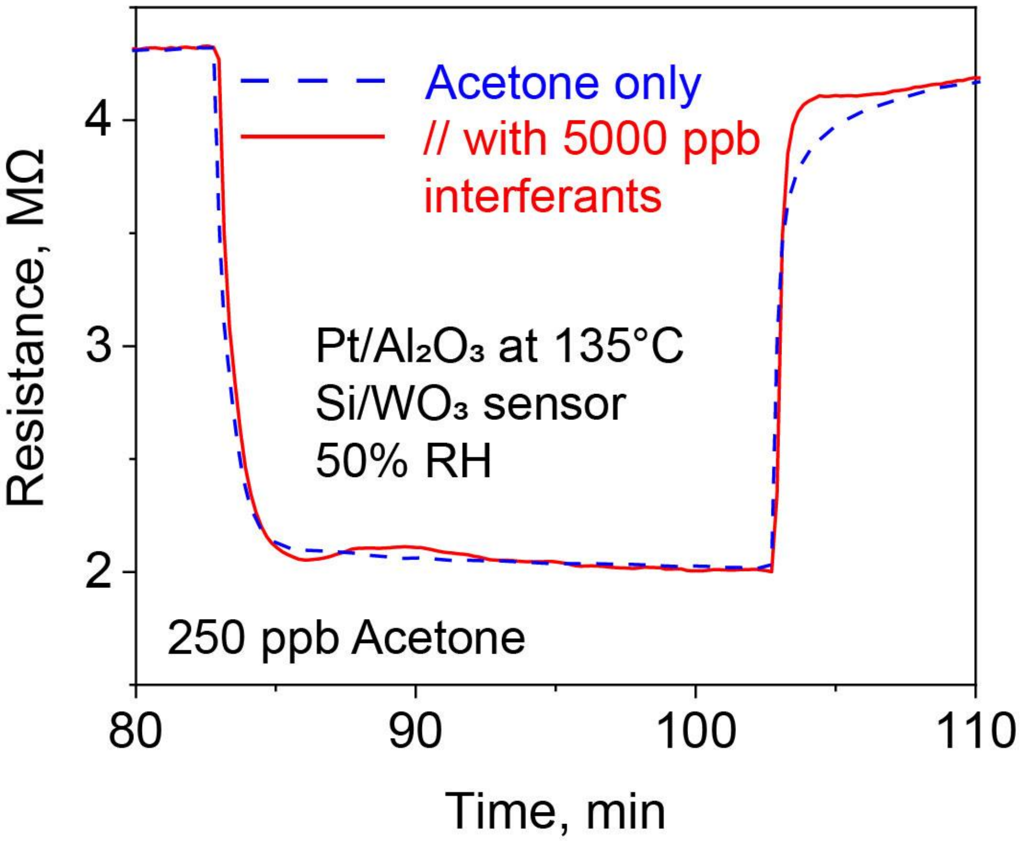

Figure S1: (a) Magnification from Figure 3a of the sensor response to 250 ppb of acetone without (acetone only, blue dashed line) and with 5000 ppb of interferants (ammonia, isoprene, ethanol, CO and H_2, each 1000 ppb, red solid line).

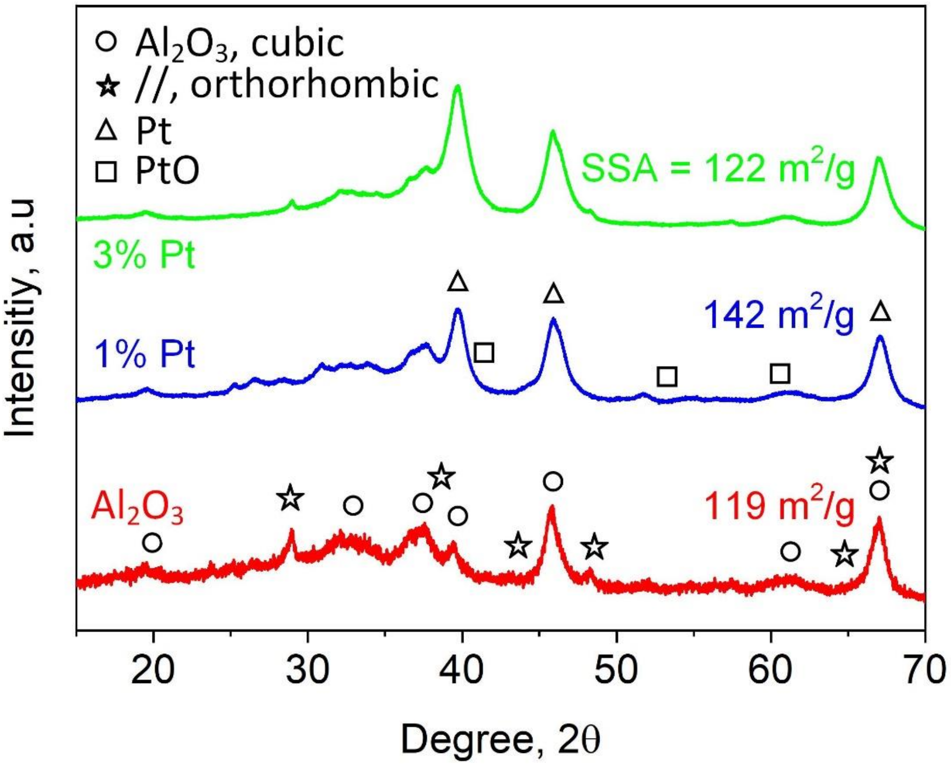

Figure S2: XRD patterns of the pure Al_2O_3 (red), 1 (blue) and 3 mol% Pt/Al_2O_3 (green) with reference peaks for cubic Al_2O_3 (circles), orthorhombic Al_2O_3 (stars), cubic Pt (triangles) and tetragonal PtO (squares). The specific surface areas (SSA) are indicated.

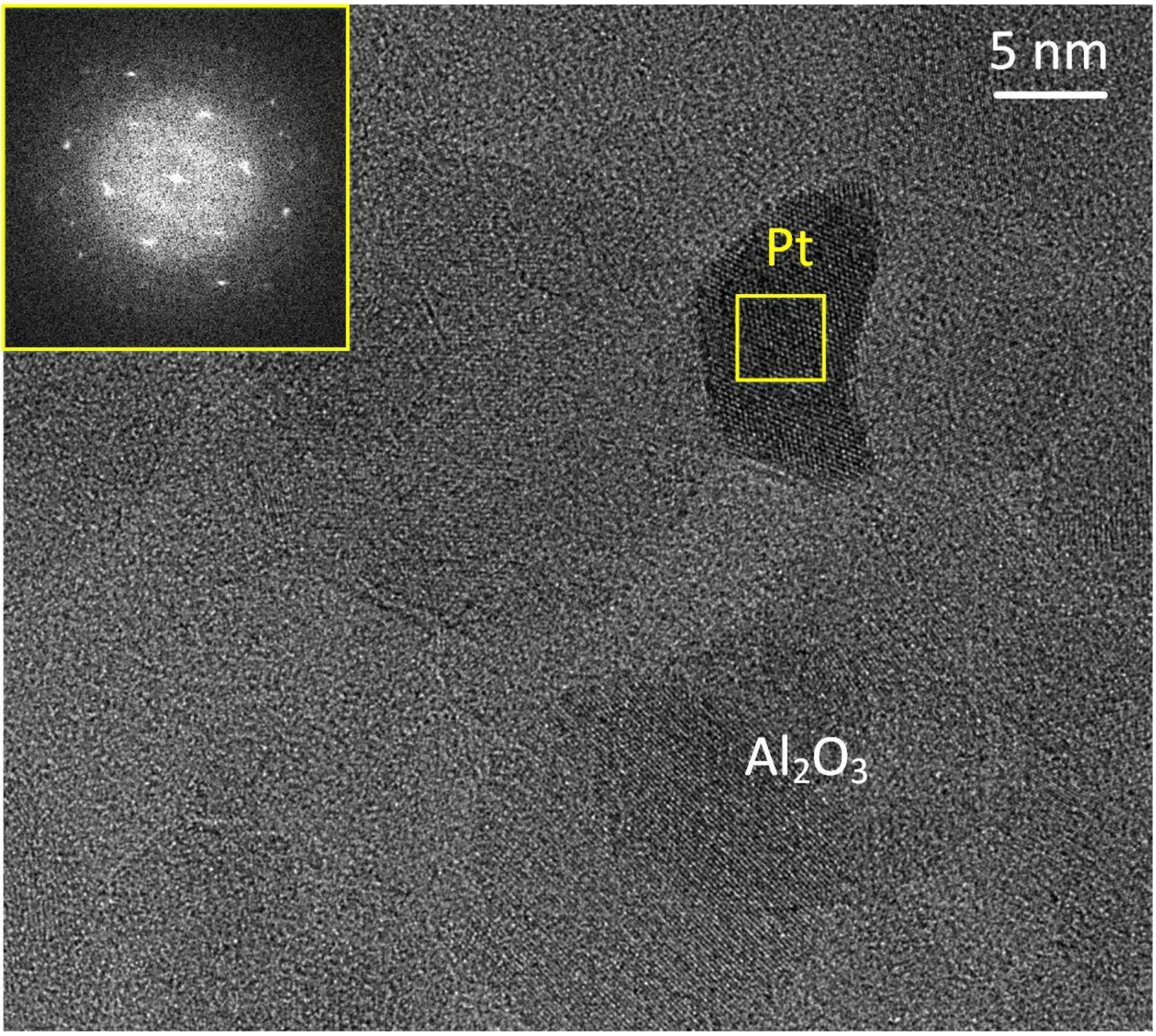

Figure S3: HRTEM image of a highly crystalline Pt particle on the Al_2O_3 support after annealing. The Pt (78 u) particle appears darker due to its larger atomic number than Al (13 u) resulting in higher scattering contrast. The crystallinity of this particle is confirmed by the spots appearing in the Fourier transform (inset).

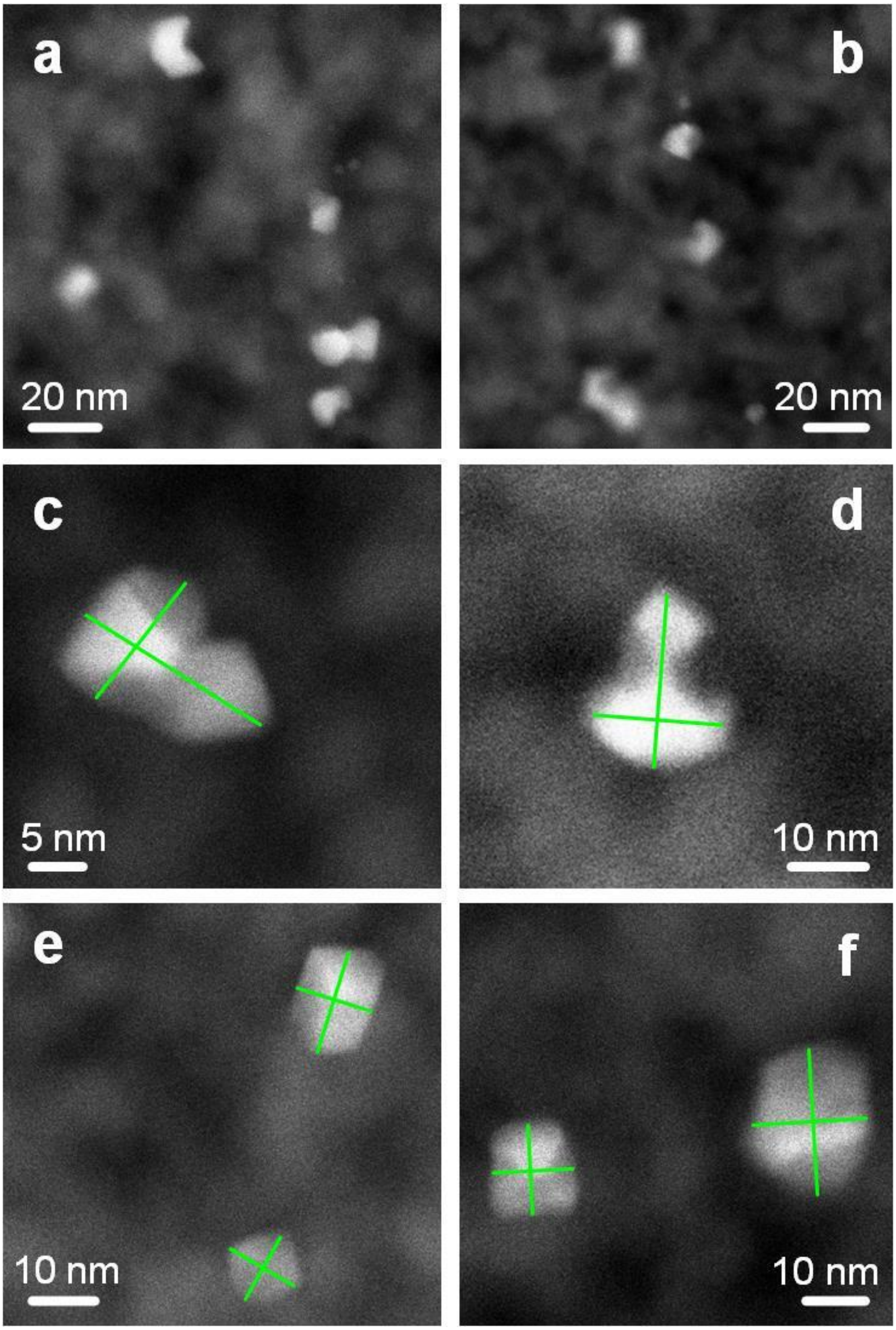

Figure S4: Size determination of Pt clusters/particles with HAADF-STEM: Overview of Pt clusters on Al_2O_3 in (a) and (b). The particle size is determined as the average of the longest diameter and its perpendicular one (c– f). Aggregation by sintering is visible (c, d).

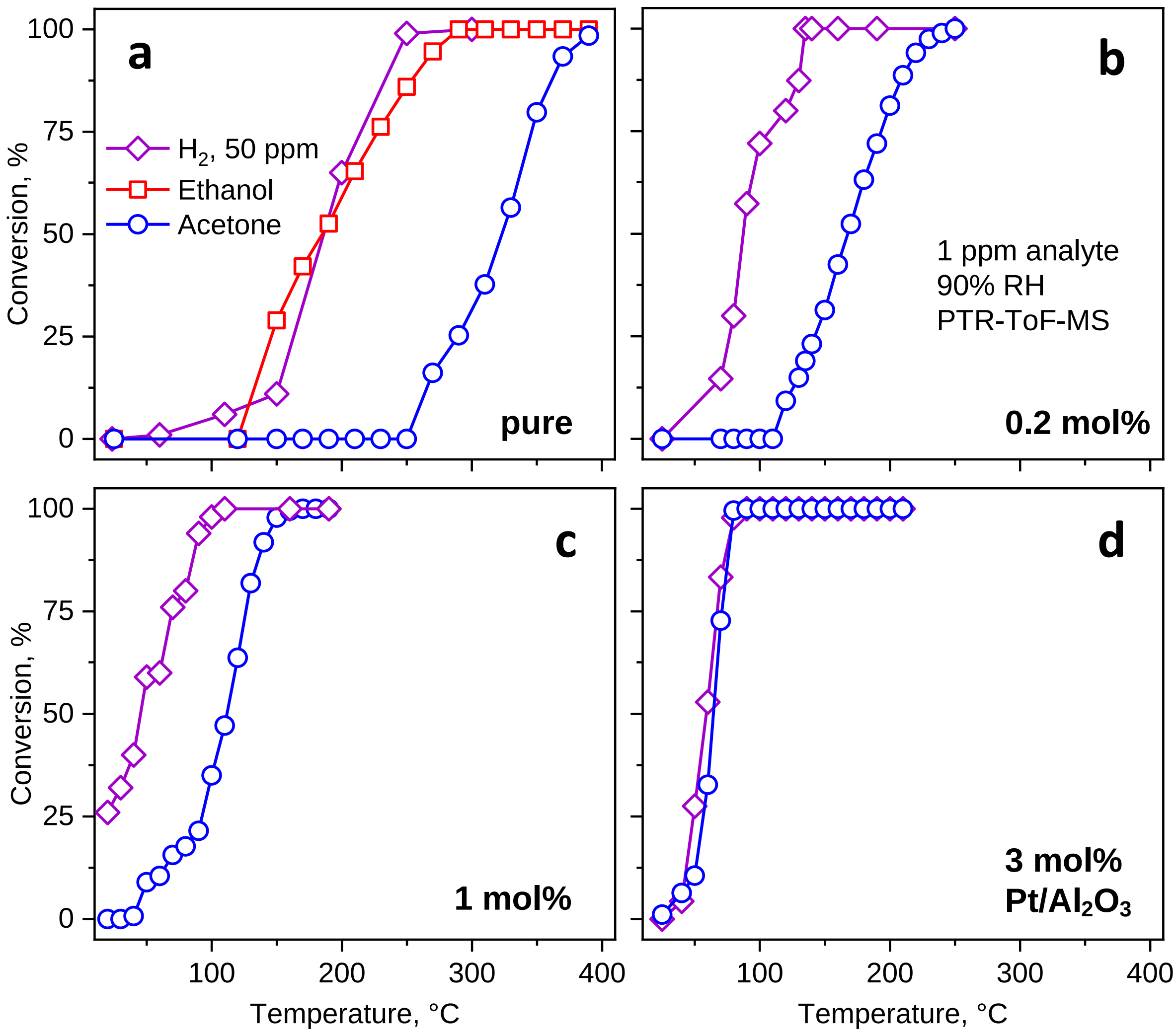

Figure S5: Catalytic conversion of 1 ppm acetone (circles), ethanol (squares) and 50 ppm H_2 (diamonds) on pure (a), 0.2 (b), 1.0 (c) and 3.0 mol% Pt/Al_2O_3 (d**)** at 90% RH as a function of catalytic packed bed temperature. Acetone and ethanol concentrations were measured by PTR-ToF-MS, while a QuinTron Breath Tracker was used for H_2.

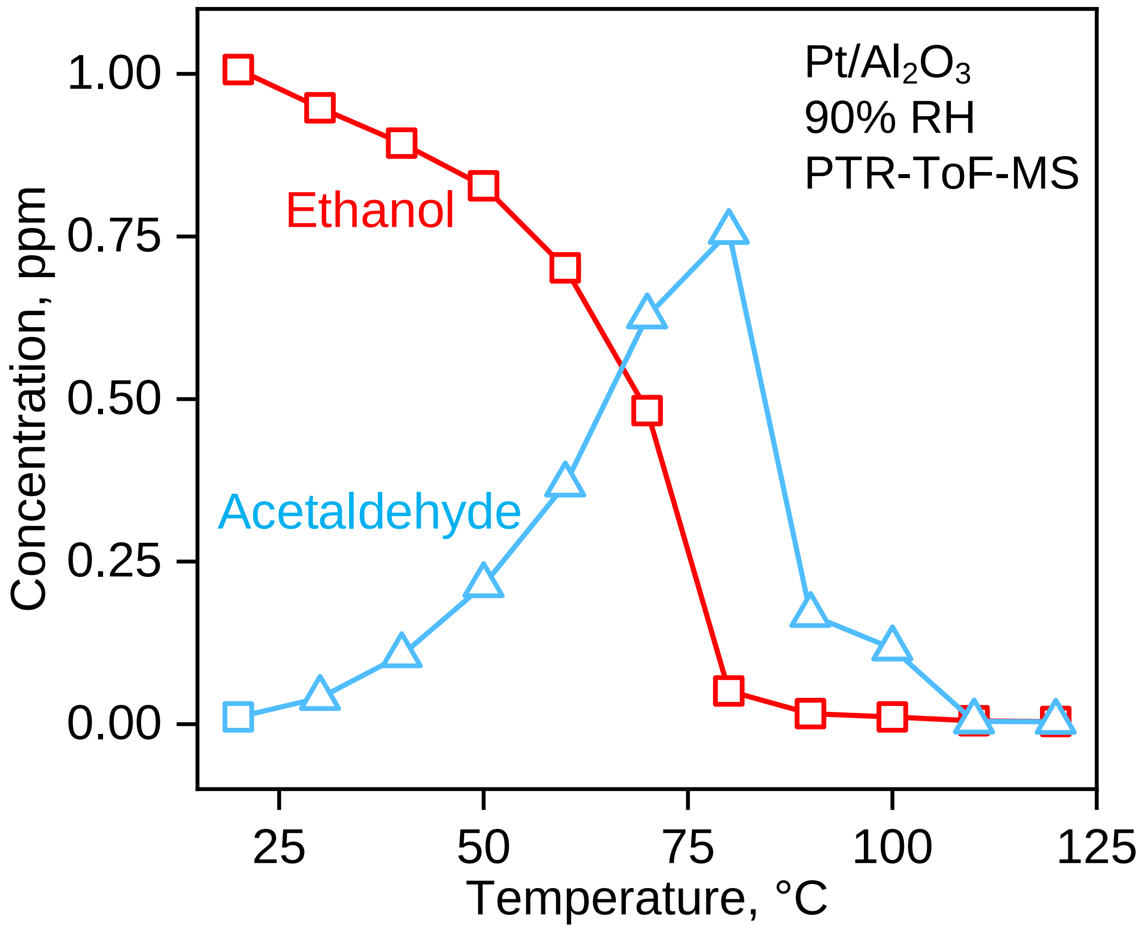

Figure S6: Catalytic conversion of 1 ppm ethanol (squares) and formation of acetaldehyde (triangles) on 0.2 mol% Pt/Al_2O_3 at 90% RH as a function of temperature. Concentrations were measured with PTR-ToF-MS at the catalyst outlet.

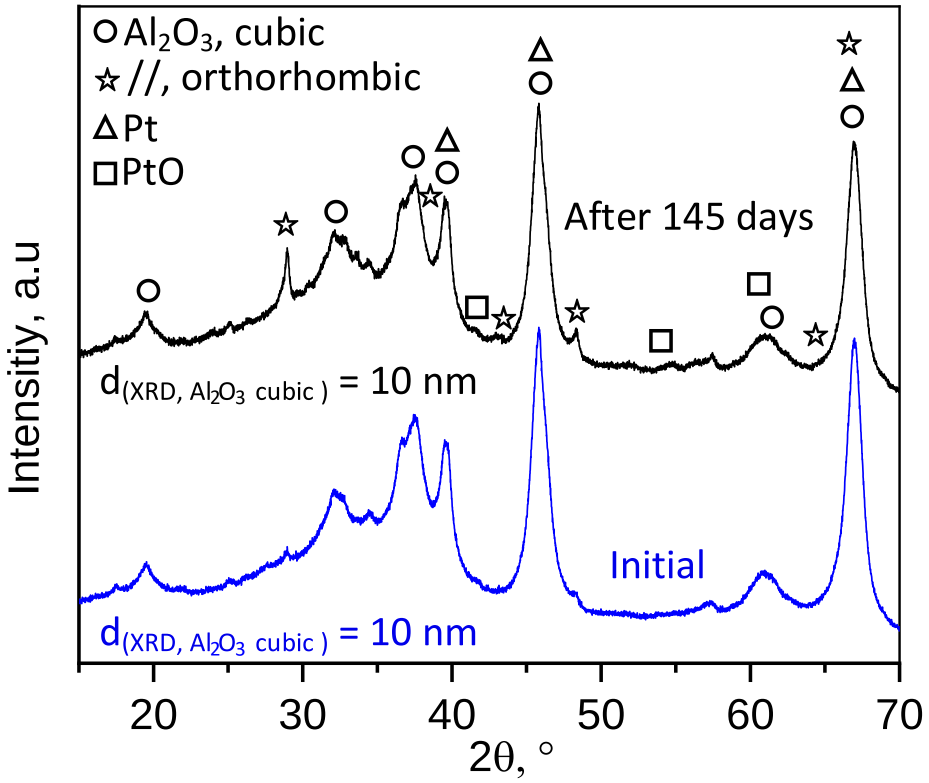

Figure S7: XRD patterns of the 0.2 mol% Pt/Al_2O_3 powders before (blue) and after 145 days (black) of testing. Reference peaks for cubic Al_2O_3 (circles), orthorhombic Al_2O_3 (stars), Pt (triangles) and tetragonal PtO (squares) are indicated together with the cubic Al_2O_3 crystal sizes (d_{XRD}).
